# Capsaicin Enhances the Drug Sensitivity of Cholangiocarcinoma through the Inhibition of Chemotherapeutic-Induced Autophagy

**DOI:** 10.1371/journal.pone.0121538

**Published:** 2015-05-01

**Authors:** Zai-Fa Hong, Wen-Xiu Zhao, Zhen-Yu Yin, Cheng-Rong Xie, Ya-Ping Xu, Xiao-Qin Chi, Sheng Zhang, Xiao-Min Wang

**Affiliations:** 1 Post-Graduate College, Fujian Medical University, Fuzhou, China; 2 Department of Hepatobiliary Surgery, Liver Disease Center of Xiamen Traditional Hospital, Xiamen, China; 3 Department of Hepatobiliary Surgery, Zhongshan Hospital Xiamen University, Xiamen, China; 4 Union Clinical Medical College, Fujian Medical University, Fuzhou, China; Zhongshan Hospital Fudan University, CHINA

## Abstract

Cholangiocarcinoma (CCA), a devastating cancer with a poor prognosis, is resistant to the currently available chemotherapeutic agents. Capsaicin, the major pungent ingredient found in hot red chili peppers of the genus Capsicum, suppresses the growth of several malignant cell lines. Our aims were to investigate the role and mechanism of capsaicin with respect to the sensitivity of CCA cells to chemotherapeutic agents. The effect of capsaicin on CCA tumor sensitivity to 5-fluorouracil (5-FU) was assessed in vitro in CCA cells and in vivo in a xenograft model. The drug sensitivity of QBC939 to 5-FU was significantly enhanced by capsaicin compared with either agent alone. In addition, the combination of capsaicin with 5-FU was synergistic, with a combination index (CI) < 1, and the combined treatment also suppressed tumor growth in the CCA xenograft to a greater extent than 5-FU alone. Further investigation revealed that the autophagy induced by 5-FU was inhibited by capsaicin. Moreover, the decrease in AKT and S6 phosphorylation induced by 5-FU was effectively reversed by capsaicin, indicating that capsaicin inhibits 5-FU-induced autophagy by activating the phosphoinositide 3-kinase (PI3K)/protein kinase B (AKT)/mammalian target of rapamycin (mTOR) pathway in CCA cells. Taken together, these results demonstrate that capsaicin may be a useful adjunct therapy to improve chemosensitivity in CCA. This effect likely occurs via PI3K/AKT/mTOR pathway activation, suggesting a promising strategy for the development of combination drugs for CCA.

## Introduction

Cholangiocarcinoma (CCA), the second most common primary hepatobiliary cancer, originates from the neoplastic transformation of epithelial cells[1]. Morbidity and mortality rates for CCA have been increasing rapidly worldwide. However, chemotherapy and radiotherapy are relatively ineffectual because of multi-drug resistance (MDR), and the results of surgical resection tend to be disappointing due to recurrence[2]. Therefore, an effective therapeutic strategy to cure this lethal tumor is desperately needed.

MDR remains one of the most pressing problems in the management of cancer patients because most patients eventually die because their disseminated cancer has become resistant to all available drugs[3, 4]. A large body of research has focused on the mechanism of MDR for the particular biological behaviors of CCA cells and has shown that many factors contribute to tumor MDR, including upregulation of drug transporters[5], avoidance of apoptosis[6], and unbalanced glucose metabolism[7] as well as increased autophagy[8, 9].

Capsaicin (8-methyl-N-vanillyl-6-noneamide), the major pungent ingredient found in hot red chili peppers of the genus Capsicum, suppresses several malignant cell lines, including human hepatoma carcinoma[10], tongue cancer[11], colon cancer[12], breast cancer[13], and glioma[14], among others[15]. Studies of the underlying mechanisms have shown that capsaicin interferes with transcriptional activation by nuclear factor-κB (NF-κB) and activator protein-1 (AP-1), resulting in the negative regulation of cell survival, adhesion, inflammation, differentiation, and growth in various cell types[15]. Although many food-derived drugs, such as capsaicin, exert antiproliferative effects on tumors, the concentrations required are often prohibitive for these compounds to be considered viable treatment options[16, 17]. A more rational approach is to study the efficacy of these compounds as adjunct therapies to existing chemotherapeutic strategies. However, the effects of capsaicin on the chemotherapy sensitivity of CCA have not been explored.

In this study, we sought to investigate the role and mechanism of capsaicin in improving the sensitivity of CCA cells to common chemotherapeutics. We found that capsaicin suppressed 5-FU-induced autophagy via activation of the phosphoinositide 3-kinase (PI3K)/protein kinase B (AKT)/mammalian target of rapamycin (mTOR) pathway in CCA cells, which rendered the cells more susceptible to 5-FU both in vitro and in vivo. Our results may provide additional insight into the potential synergistic effects of capsaicin in combination with chemotherapeutics on CCA cells and facilitate the continued development of new anticancer treatments.

## Material and Methods

### Reagents and antibodies

5-Fluorouracil (5-FU), vincristine sulfate (VCR), cisplatin (cis-diamminedichloroplatinum(II), CDDP), capsaicin, 3-(4,5-dimethylthiazol-2-yl)-2,5-diphenyl tetrazolium bromide (MTT), 3-methyladenine (3-MA), and rapamycin (RARA) were purchased from Sigma-Aldrich (Indianapolis, IN, USA). Antibodies against glyceraldehyde 3-phosphate dehydrogenase (GAPDH), phospho-AKT(S473), AKT, phospho-S6(S235/236), S6, and microtubule-associated protein 1 light chain 3 (LC3) were all were purchased from Abcam, Ltd. (Cambridge, UK). Goat anti-rabbit and anti-mouse secondary antibodies conjugated to horseradish peroxidase were purchased from Thermo Scientific Pierce Co., Ltd. (Rockford, IL, USA). An Annexin V-fluorescein isothiocyanate (FITC)/propidium iodine (PI) double-staining apoptosis detection kit and TdT-mediated dUTP nick-end labeling (TUNEL) kit were purchased from Roche Bio (Basel, Switzerland).

### Cell culture

Three human CCA cell lines, QBC939, SK-ChA-1, and MZ-ChA-1, were obtained from the Cell Bank of the Chinese Academy of Sciences (Shanghai, China). The cells were cultured in Roswell Park Memorial Institute (RPMI)-1640 medium supplemented with 10% fetal bovine serum (FBS), 100 U/mL ampicillin, and 100 U/mL streptomycin sulfate at 37°C in a humidified atmosphere with 5% CO_2_. The cells were treated with culture media containing various concentrations of drugs at 24 h after seeding.

### Cell proliferation assay

The cells were seeded at 3 × 10^3^ per well in 96-well plates and grown overnight. After treatment with a series of concentrations of 5-FU, CDDP, VCR, and capsaicin (alone or in combination) for 24 h, 20 μL MTT (5 mg/mL) was added to each well, and the cells were cultured for another 4 h at 37°C. The formazan crystals formed were dissolved in dimethyl sulfoxide (DMSO), and the absorbance was measured at 490 nm using an enzyme-linked immunosorbent assay (ELISA) microplate reader. Each experiment was performed in triplicate and repeated at least three times.

### Drug combination experiment

The effect of the drug combination was analyzed using the combination index (CI) method, as defined by the following equation: CI = (OD490)AB/[(OD490)A + (OD490)B], where (OD490)AB was the absorbance of the group with combined treatment with drugs A and B, and (OD490)A and (OD490)B were the absorbance for the treatment groups with drugs A and B alone, respectively. CI values >1 indicated antagonism, CI values = 1 indicated additivity, CI values<1 indicated synergy, and CI values <0.7 indicated significant synergy. Each CI ratio represented here was the mean value derived from at least three independent experiments.

### Cell apoptosis detection

The apoptosis of CCA cells in vitro was detected by Annexin V-FITC/PI staining. Both floating and adherent cells were collected after treatment (24 h) and were washed twice with ice-cold phosphate-buffered saline (PBS). The cells were then resuspended in 500 μL of binding buffer and stained with Annexin V-FITC and PI according to the manufacturer’s instructions using the Annexin V-FITC/PI Apoptosis Detection Kit. The signal was detected using a FACScan (FACStation; BD Biosciences, San Jose, CA, USA) with BD FACSCalibur and CellQuest software (using a Macintosh computer; Apple Computer, Cupertino, CA, USA). Apoptosis in the CCA cells in vivo was detected by TUNEL analysis according to the kit manufacturer’s instructions.

### In vivo tumor xenograft studies

Nude mice (BALB/c, SPF grade, 4–5 weeks of age) were injected subcutaneously with 100 μL of cells (2×106) and sacrificed at day 21 after cell injection. The tumor volumes were determined according to the following formula: A×B^2^/2, where A is the largest diameter and B is the perpendicular diameter. The drug susceptibility experiments were divided into four groups (PBS, 5-FU, capsaicin, and 5-FU+capsaicin). The dosage of 5-FU was 60 mg/kg/d, and the dosage of capsaicin was 150 mg/kg/d; the mice were treated at the 8st day after post-transplantation. All of the manipulations involving living mice were approved by the Animal Care and Use Committee of the Affiliated Zhongshan Hospital of Xiamen University, and all efforts were made to minimize suffering.

### Western blot analysis

Cell lysates containing equal amounts of protein were separated on 10% sodium dodecyl sulfate (SDS)-polyacrylamide gels and electrotransferred onto polyvinylidene difluoride (PVDF) membranes. The membranes were blocked in 5% milk in PBS with Tween-20 (PBST) (NaCl 137 mmol/L, KCl 2.7 mmol/L, Na_2_HPO_4_ 10 mmol/L, KH_2_PO_4_ 2 mmol/L, 0.05% Tween-20) for 1 h and then incubated with the indicated primary antibody overnight at 4°C. After three washes in PBST, the blots were then incubated with a horseradish peroxidase-conjugated secondary antibody and visualized by chemiluminescence. GAPDH was used as an internal control.

### Statistical analysis

The data are expressed as the mean ± standard error of the mean (SEM) for given samples and evaluated by Student’s t test or a one-way analysis of variance (ANOVA) using Statistical Package for Social Science (SPSS) 16 statistical software (SPSS Inc., Chicago, IL, USA). Each assay was repeated in triplicate. The level of significance was set at *P*<0.05.

## Results

### CCA cells exhibit MDR to chemotherapeutic agents, including capsaicin

**Fig 1:**
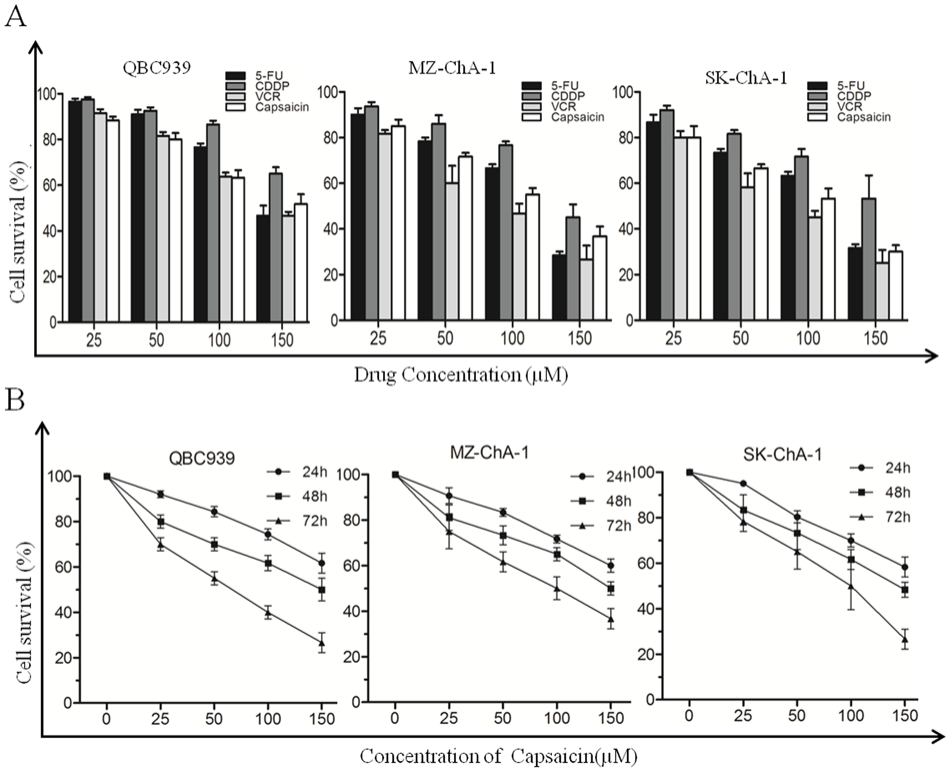
The susceptibility of CCA cells to chemotherapeutics and capsaicin. (A) Cytotoxic effects of common chemotherapeutics, including capsaicin, on three CCA cell lines. Cell viability was measured by MTT assay after treatment for 48 h. (B) Three CCA cell lines were treated with different concentrations of capsaicin for 24 h, 48 h, and 72 h. Cell viability was measured by MTT assay.

An MTT assay was performed to evaluate the effects of chemotherapeutic agents on three human CCA cell lines, QBC939, SK-ChA-1, and MZ-ChA-1. As previously reported[18], the CCA cells were found to be resistant to common chemotherapeutic agents such as VCR, 5-FU, and CDDP and showed slight cytotoxic effects only at high concentrations (Fig 1A). We also found that the 50% inhibition concentrations (IC_50_) of capsaicin for QBC939, SK-ChA-1, and MZ-ChA-1 were all high (Fig 1B), though capsaicin could reduce the viability of the CCA cells in a dose- and time-dependent manner. The IC_50_ values for the cell lines when incubated for 24 h, 48 h, and 72 h, respectively, were as follows: QBC939, 173.3 ±1.34, 104.36 ± 1.07, and 52.06 ± 1.15 μM; MZ-ChA-1, 219.04 ± 1.19, 133.69 ± 1.06, and 79.30 ± 1.53 μM; and SK-ChA-1, were 189.04 ± 1.39, 112.69 ± 1.06, and 75.23 ± 1.33 μM. These results suggested that CCA cells exhibited MDR to VCR, 5-FU, and CDDP as well as to capsaicin.

### Capsaicin enhances the drug sensitivity of CCA cells to 5-FU in vitro

**Fig 2:**
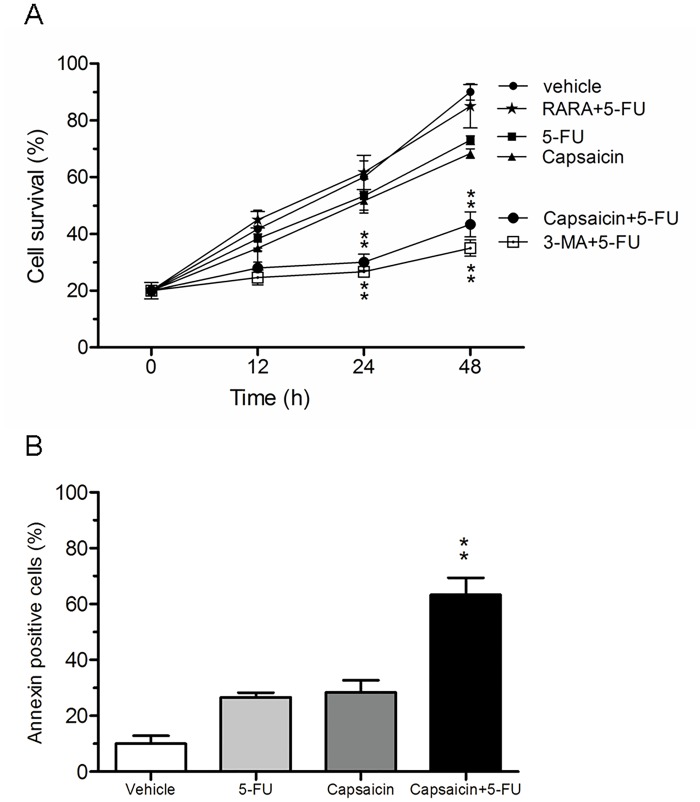
The effects of capsaicin in combination with 5-FU on QBC939 cells. (A) Co-treatment with capsaicin and the autophagy inhibitor 3-MA enhanced the cytotoxic effect of 5-FU on QBC939 cells. Cell viability was measured by MTT assay after 48 h. Three drug combinations (RARA, 3-MA, and capsaicin) were compared to 5-FU at each treatment time, respectively. (B) Co-treatment with capsaicin increased 5-FU-induced apoptosis. Apoptosis was detected by Annexin V-FITC/PI staining after 48 h. 5-FU (40 μM), RARA (2 mM), 3-MA (1 mM), capsaicin (60 μM). (Capsaicin + 5-FU) vs. 5-FU. * *P*<0.05, ** *P*<0.01.

Initially, we used a fixed low concentration (60 μM, lower than the 48 h IC_50_) of capsaicin and studied the effect of the combination with 5-FU (40 μM, lower than the 48 h IC_50_) on the CCA QBC939 cell line. The results of the drug combination experiment showed that a low concentration of capsaicin had a synergistic effect on the antiproliferative actions of 5-FU from the 24 h timepoint (Fig 2A). The effects of capsaicin in combination with 5-FU on QBC939 cells are further detailed in Table 1. The IC_50_ of QBC939 cells for 5-FU in combination with capsaicin (20, 40, 80 μM) was significantly decreased from 126 μM to 35 μM, and significant synergy with capsaicin was found at 40 μM. Furthermore, an analysis of apoptosis using Annexin V-FITC double staining showed that capsaicin treatment (40 μM) increased the susceptibility of CCA QBC939 cell lines to 5-FU-induced apoptosis (Fig 2B). These results indicate that the sensitivity of QBC939 cells to 5-FU was enhanced by capsaicin.

**Table 1:** Effect of capsaicin in combination with 5-FU on QBC939 cells.

Capsaicin (μM)	5-FU (μM)	A_490_	CI	IC_50_ (μM)
0	0	1.075 ± 0.063		
20	0	1.024 ± 0.095		
40	0	1.017±0.068		
80	0	0.702±0.065		
0	20	0.914±0.053		
0	40	0.852±0.085		126
0	80	0.758±0.092		
20	20	0.892±0.046	1.025^a^	
20	40	0.871±0.055	1.000^b^	102
20	80	0.752±0.028	0.927^c^	
40	20	0.733±0.095	0.848^c^	
40	40	0.558±0.077	0.692^d^	67
40	80	0.346±0.081	0.482^d^	
80	20	0.492±0.065	0.824^c^	
80	40	0.403±0.084	0.724^c^	35
80	80	0.352±0.039	0.711^c^	

^a^, antagonism, CI >1;

^b^, additivity, CI = 1;

^c^, synergy, CI <1;

^d^, significant synergy, CI <0.7.

### Capsaicin renders CCA cells more susceptible to 5-FU-induced apoptosis in vivo

**Fig 3:**
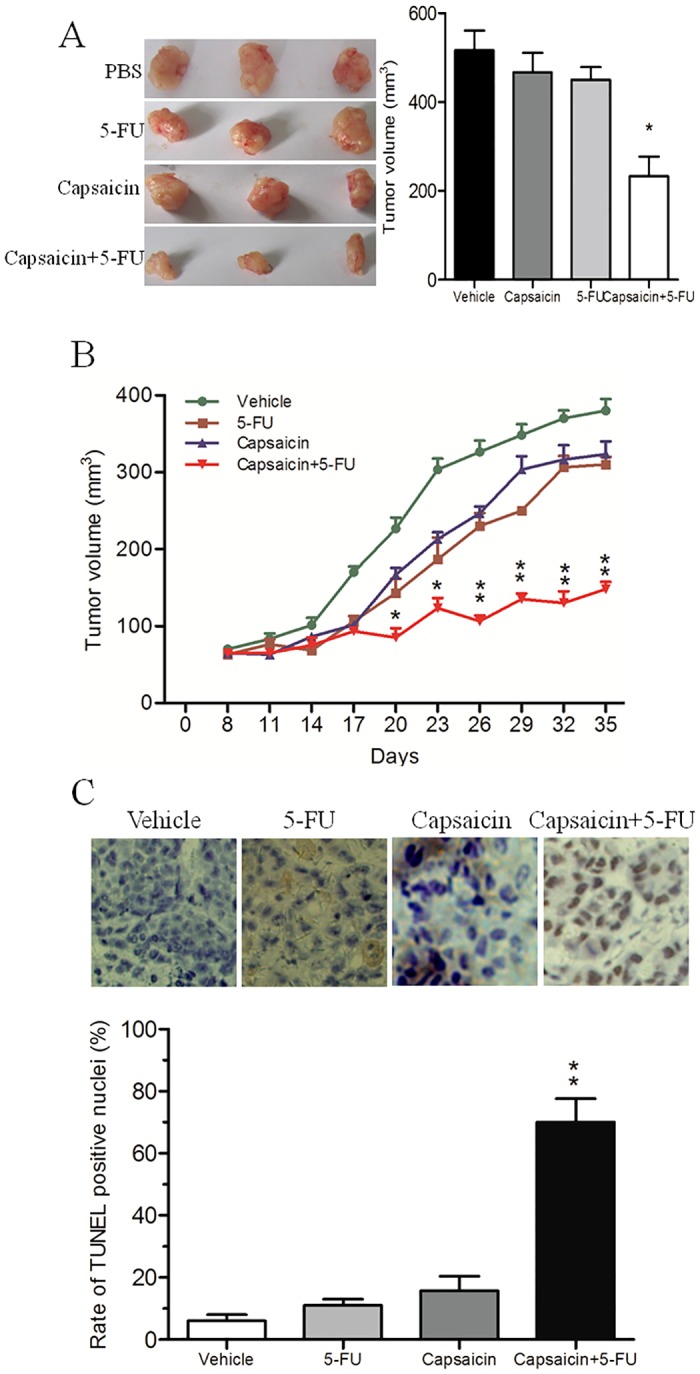
The cytotoxic effect of 5-FU in combination with capsaicin in vivo. (A) Combination treatment of 5-FU and capsaicin significantly reduced the xenografted CCA tumor volume. (B) Combination treatment with 5-FU and capsaicin effectively suppressed CCA tumor growth. (C) Capsaicin increased 5-FU-induced apoptosis in vivo. Apoptosis was detected by TUNEL assay. * *P*<0.05, ** *P*<0.01.

Next, we investigated whether capsaicin would enhance apoptosis in a mouse xenograft tumor model. Although the administration of 5-FU or capsaicin alone did not affect tumor growth compared with vehicle treatment (Fig 3A), treatment with capsaicin in combination with 5-FU resulted in a significant decrease in tumor volume (Fig 3A) and a stronger inhibition of tumor growth (Fig 3B). The results of a cell apoptosis evaluation by TUNEL assay were consistent with the in vitro data: tumors treated with a combination of capsaicin and 5-FU exhibited a significant increase in apoptosis compared with tumors treated with 5-FU or capsaicin alone (Fig 3C). These results demonstrated that a combination therapy of capsaicin and 5-FU effectively inhibited CCA tumor progression.

### Capsaicin inhibits 5-FU-induced autophagy by activating the PI3K/AKT/mTOR pathway in CCA cells

**Fig 4:**
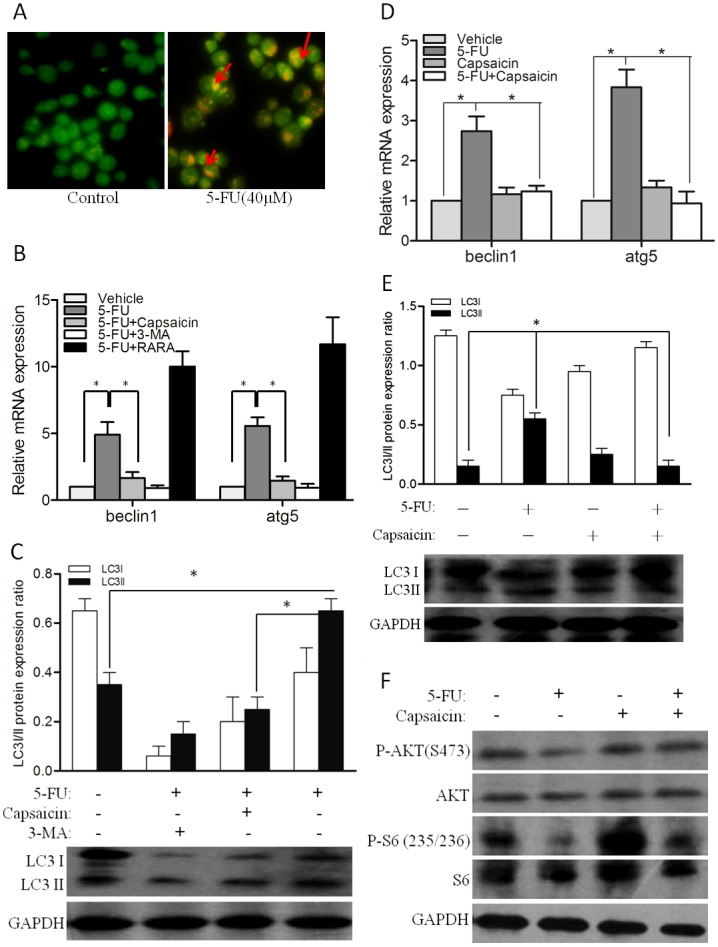
5-FU-induced autophagy was inhibited by capsaicin. (A) Acridine orange staining showing lysosomal (red or orange) staining in the cells in the 5-FU treatment group. (B and D) The expression of beclin1 and atg5 mRNA was detected by real-time polymerase chain reaction (PCR) in vitro (B) and in vivo (D). (C and E) Western blot analysis of LC I/II protein expression exposed to different treatments in vitro (C) and in vivo (E). (F) Western blot analysis of the AKT/mTOR pathway in QBC939 cells exposed to different treatments after 24 h. GAPDH was used as a loading control. 5-FU (40 μM), capsaicin (40 μM), control/vehicle (PBS). * *P*<0.05, ** *P*<0.01.

Previous studies have indicated that autophagy is induced following treatment with cytotoxic agents, which induces autophagic drug resistance to cancer therapies[8]. In our study, we first found that the autophagy inhibitor 3-MA could enhance the susceptibility of QBC939 cells to 5-FU, and inversely, the autophagy activator RARA could render the QBC939 cells resistant to 5-FU (Fig 2A). Moreover, we found that an increase in acridine orange staining intensity was readily apparent after 5-FU treatment, with the expression of beclin1 and atg5 both significantly elevated (Fig 4A and 4B), and the level of LC3II protein was increased by 5-FU treatment (Fig 4C). In addition, the expression patterns of autophagy associated genes (beclin1, atg5, and LC3II) in vivo were also elevated (Fig 4D and 4E). These observations indicated that the MDR of CCA cells to 5-FU might be associated with autophagy.

Because capsaicin renders CCA cells more susceptible to 5-FU in vitro and in vivo, we hypothesized that capsaicin could inhibit 5-FU-induced autophagy. It is noteworthy that the upregulation of both beclin1 and atg5 could be reversed by capsaicin and the autophagy inhibitor 3-MA (Fig 4B–4D). Furthermore, there was a decrease in LC3II protein levels after co-treatment with capsaicin or 3-MA compared with vehicle or 5-FU treatment alone (Fig 4C–4E). The AKT/mTOR pathway is the key regulator of autophagy[8]. In a molecular mechanism study, we found that capsaicin effectively increased the phosphorylation levels of S6 (S235/236) and AKT (S473), particularly reversing the decreased in p-S6 and p-AKT induced by 5-FU (Fig 4F). These findings suggested that capsaicin inhibited 5-FU-induced autophagy by activating the AKT/mTOR pathway in CCA cells.

## Discussion

Currently, the molecular mechanism of MDR is still unclear, and moreover, there is no effective targeted therapy for CCA patients. Here, we provide in vitro and in vivo evidence that co-treatment with capsaicin enhanced the antiproliferative effects of 5-FU on CCA cells through the inhibition of 5-FU-induced autophagy, suggesting that utilizing capsaicin as an adjunct therapy may decrease CCA chemoresistance.

Among cancers, CCA is one of the most resistant to treatment, and the treatment options are limited; conventional chemotherapy and radiation therapy to date have been notably ineffective in improving long-term survival[2]. Our data are consistent with previous studies showing that CCA cells are resistant to common chemotherapeutic agents, including VCR, 5-FU, and CDDP[18]. Although capsaicin has long been suggested as an effective, naturally occurring chemopreventive agent that acts to induced apoptosis and cell cycle arrest[12, 19], we here showed that capsaicin alone requires a high IC_50_ concentration to inhibit the proliferation of three CCA cell lines. Given the rising incidence of CCA and the paucity of effective treatments[1], there is much hope for targeted therapies and promising agents, including agents that act as an adjunct therapy.

An increasing number of studies have been published regarding the efficacy of food- or plant-derived compounds such as resveratrol, tannic acid, escin, and green-tea polyphenols as adjunct therapies to the currently available chemotherapeutic strategies[17, 18, 20, 21]. Capsaicin, an important pungent ingredient extracted from chili peppers of the genus Capsicum, is able to inhibit the growth of various types of cancer cells, such as human hebpatoma carcinoma, colon cancer, breast cancer and neuroblastoma cells[10, 12]. However, the effect of capsaicin in comination with chemotherapeutic agents has remained unclear. Drug combinations are widely used in cancer treatment for over half a century. It is a rational and efficient strategy to increase therapeutic efficacy [22]. Here, we demonstrated a novel role for capsaicin in combination with 5-FU, which enhanced the susceptibility of QBC939 cells in vitro and in vivo. As known, synergistic combination may increase the therapeutic efficacy while decrease toxicity and overcome resistance [23]. In our drug combination experiment, capsaicin reversed the MDR of CCA cells through promoting the apoptosis and reduced the IC50 of 5-FU. Therefore, this capsaicin combination might be capable of reducing the perniciousness of drug resistance in CCA.

Autophagy is an evolutionarily conserved catabolic pathway where cells deliver their own cytoplasmic material and/or organelles to lysosomes for degradation[9]. Accumulating evidence shows that the increased level of autophagy induced by chemotherapy contributes to tumor MDR[24]. Autophagy serves as an adaptive stress response in tumor cells that facilitates their survival in settings of increased metabolic demand, hypoxic microenvironments, or cancer therapies[25]. Stress-induced autophagy in tumor cells is predominantly cytoprotective and maintains cell survival, which ultimately contributes to MDR in various types of tumors[9, 26]. In the current study, 5-FU treatment alone induced CCA cells autophagy in vitro and in vivo, and the autophagy activator RARA could render the CCA cells more resistant to 5-FU. Drug-induced autophagy may have facilitated the survival of the tumor cells and contributed MDR in the CCA cells[27]. On the basis of previous study that the blockade of cancer cell autophagy is emerging as a novel approach to enhance the efficiency of chemotherapy in cancer treatment[8, 28]. Interestingly, capsaicin inhibited 5-FU-induced autophagy and enhanced the drug susceptibility of CCA cells. Therefore, our data showed that autophagy is activated in the process of CCA chemotherapy, and inactivation of autophagy may facilitate the drug-induced apoptosis and enhance chemotherapy sensitivity.

There is a convincing evidence that the PI3K/Akt/mTOR pathway represents the major negative regulator of autophagy[29]. Selective regulation of PI3K/AKT/mTOR signaling represents a promising approach for cancer and might prove useful when combined with other drugs[30]. Capsaicin could activated the PI3K/Akt/mTOR pathway, and the decrease of Akt and pS6 kinase phosphorylation induced by 5-FU was returned after treatment with capsaicin. Thus, the activation of PI3K/Akt/mTOR pathway by capsaicin inhibited 5-FU-induced autophagy.

Taken together, our study demonstrates that capsaicin inhibits 5-FU-induced autophagy by activating the AKT/mTOR pathway and rendering CCA cells more susceptible to 5-FU-induced apoptosis. This study might offer a possible molecular basis for the further development of combinations of capsaicin and common agents as a novel therapeutic approach for CCA patients.
